# Expression of Procyclin mRNAs during Cyclical Transmission of *Trypanosoma brucei*

**DOI:** 10.1371/journal.ppat.0010022

**Published:** 2005-11-04

**Authors:** Simon Urwyler, Erik Vassella, Jan Van Den Abbeele, Christina Kunz Renggli, Pat Blundell, J. David Barry, Isabel Roditi

**Affiliations:** 1 Institute of Cell Biology, University of Bern, Switzerland; 2 Department of Parasitology, Prince Leopold Institute of Tropical Medicine, Antwerp, Belgium; 3 Swiss Tropical Institute, Basel, Switzerland; 4 Wellcome Centre for Molecular Parasitology, University of Glasgow, United Kingdom; Washington University School of Medicine, United States of America

## Abstract

*Trypanosoma brucei*, the parasite causing human sleeping sickness, relies on the tsetse fly for its transmission. In the insect, EP and GPEET procyclins are the major surface glycoproteins of procyclic (midgut) forms of the parasite, with GPEET predominating in the early procyclic form and two isoforms of EP in the late procyclic form. EP procyclins were previously detected on salivary gland trypanosomes, presumably epimastigotes, by immunoelectron microscopy. However, no procyclins could be detected by mass spectrometry when parasites were isolated from infected glands. We have used qualitative and quantitative RT-PCR to analyse the procyclin mRNAs expressed by trypanosomes in the tsetse midgut and salivary glands at different time points after infection. The coding regions of the three EP isoforms (EP1, EP2 and EP3) are extremely similar, but their 3′ untranslated regions contain unique sequences that make it possible to assign the cDNAs amplified by this technique. With the exception of EP2, we found that the spectrum of procyclin mRNAs expressed in the midgut mirrors the protein repertoire of early and established procyclic forms. Surprisingly, procyclin mRNAs, including that of GPEET, are present at relatively high levels in salivary gland trypanosomes, although the proteins are rarely detected by immunofluorescence. Additional experiments using transgenic trypanosomes expressing reporter genes or mutant forms of procyclin point to a mechanism of translational or post-translational control, involving the procyclin coding regions, in salivary gland trypanosomes. It is widely accepted that *T. brucei* always has a coat of either variant surface glycoprotein or procyclin. It has been known for many years that the epimastigote form does not have a variant surface glycoprotein coat. The finding that this life cycle stage is usually negative for procyclin as well is new, and means that the paradigm will need to be revised.

## Synopsis

The tropical parasite *Trypanosoma brucei* relies on the tsetse fly for its transmission between mammals. The parasite first establishes an infection in the fly midgut then migrates and colonizes the salivary glands. A paradigm in trypanosome biology is that parasites in contact with the mammalian host are covered by variant surface glycoproteins, enabling them to evade the immune system, while all other life cycle stages are covered by repetitive glycoproteins known as procyclins.

In this publication the authors investigated the expression pattern of procyclins in all life cycle stages in infected tsetse flies, including epimastigote and metacyclic forms in the salivary glands. These stages are usually neglected as, in contrast to trypanosomes from the midgut, they cannot be cultured. The researchers showed that the vast majority of trypanosomes in the salivary glands have no procyclin coat although the levels of procyclin messenger RNAs are comparatively high. Experiments with reporter genes indicated that procyclin expression in the glands is regulated by the coding region. The lack of procyclins on salivary gland trypanosomes leads to the prediction that the epimastigote form possesses a novel surface coat that may be responsible for the tight binding of the parasite to gland tissue.

## Introduction

*Trypanosoma brucei* is a digenetic parasite that relies on tsetse flies for its transmission between mammals. The parasite causes the disease Nagana in domestic animals in sub-Saharan Africa and two subspecies, *T. b. rhodesiense* and *T. b. gambiense*, also cause human sleeping sickness. The fly (*Glossina* spp.) is the definitive host, where genetic exchange can occur, and this part of the life cycle is considerably more complex than that in the mammalian host. When a tsetse fly takes a blood meal from an animal carrying *T. brucei*, the parasites reach the midgut lumen and encounter proteases that can trigger the differentiation of the short stumpy bloodstream form to the early procyclic form [1,2]. An infection becomes established when trypanosomes migrate to the ectoperitrophic space of the gut and colonise it as late procyclic forms. It has been recognised only fairly recently that early and established procyclic forms are separate entities [3]. In a second phase, known as maturation, the parasite progresses through several more rounds of differentiation, from the mesocyclic form in the anterior midgut to a recently described asymmetrically dividing form that is thought to deliver the epimastigote form to the salivary gland [4]. Proliferating epimastigote forms that are tightly associated with the salivary gland epithelium give rise, in turn, to non-proliferating metacyclic forms that are released with the saliva. It is the metacyclic form that is capable of infecting a new mammalian host.

It is becoming apparent that the complexity of the life cycle in the fly is matched by equally complex changes at the surface of the parasite. Metacyclic forms and bloodstream forms are covered by a coat of variant surface glycoprotein (VSG) that protects the parasite from the host's immune system (reviewed in [5]). Differentiation of the bloodstream to the early procyclic form involves shedding of the VSG coat and its replacement by a new coat consisting of procyclins [6,7]. There are four different isoforms of procyclin that are named according to internal repeats, GPEET—which contains pentapeptide repeats and is highly phosphorylated—and EP1, EP2 and EP3, all of which contain glutamic acid-proline dipeptide repeats [8,9]. EP1 and EP3 are modified by N-linked carbohydrates whereas EP2 has no such glycosylation site. The individual isoforms can be distinguished by matrix-assisted laser desorption time-of-flight (MALDI-TOF) mass spectrometry [10]. We have previously used this technique to examine the procyclin repertoire of trypanosomes differentiating synchronously in culture [11] and of trypanosomes isolated from infected tsetse flies [12]. In culture, all procyclins are expressed at similar levels 3–6 h after triggering differentiation [11], during the phase in which the VSG coat is being replaced [7]. By 24 h, the cells show the characteristic profile of early procyclic forms, with high levels of GPEET and traces of EP. GPEET is phosphorylated by a membrane-associated kinase [13] that also becomes active within a few hours of triggering differentiation [3,11,14]. If glycerol is present in the medium [3] and the mitochondrial alternative oxidase is active [15], the cells continue to proliferate as early forms and express GPEET. In medium lacking glycerol, the trypanosomes differentiate to late procyclic forms within 7–9 d; this is marked by the replacement of GPEET by the glycosylated isoforms of EP (EP1 and EP3) [11] and loss of GPEET kinase activity [13]. The same sequence of events, expression of GPEET by early procyclic forms and its replacement by EP1 and/or EP3 in established procyclic forms also occurs during tsetse fly infections [12]. The repression of GPEET in late procyclic forms—both in culture and in the fly—operates through a 25 nucleotide sequence, known as the glycerol responsive element, in the 3′ untranslated region (UTR) of GPEET mRNA [15].

Almost nothing is known about procyclin expression later in the life cycle, as there is no culture system for epimastigote and metacyclic forms of *T. brucei*. EP procyclins have been detected on salivary gland trypanosomes by immunoelectron microscopy [16]. However, no procyclins could be detected by mass spectrometry when trypanosomes were isolated from the salivary glands and processed in the same way as midgut forms [12]. As an alternative to studying the proteins, we have analysed the expression and distribution of procyclin mRNAs in trypanosomes in the tsetse midgut and salivary glands at different points after infection.

## Results

### Analysis of the Procyclin mRNA Repertoire in Tsetse-Derived Trypanosomes

A possible explanation for the failure to detect procyclins by MALDI-TOF analysis of salivary gland trypanosomes [12] is that proteins that are expressed at low levels might be overlooked, particularly when post-translational modification or processing results in a range of products with different masses. We previously found this to be the case for EP procyclins in early procyclic forms; these could be detected at low levels in cultured trypanosomes [11], but not at all in midgut-derived trypanosomes because of heterogeneous cleavage by tsetse proteases [12]. As an alternative approach to ascertain if procyclins are expressed by salivary gland trypanosomes, we first used RT-PCR to assay for the presence and the relative distribution of transcripts. Total RNA was isolated from salivary glands at different time points after infection and all four types of procyclin cDNA were amplified using a single pair of primers. RNA isolated from the midguts of the same flies was used as a positive control. We assumed that the ratios of the different procyclin cDNAs would not change significantly during amplification as the mRNAs are very similar in length and sequence. The products were cloned and assigned by hybridisation with oligonucleotides specific for the 3′ UTRs of the individual EP genes or the GPEET coding region. In total, 1,584 procyclin cDNA clones were obtained from infected midguts and 897 from the salivary glands. The distribution of the different types of procyclin during the course of infection is shown in Figure 1.

**Figure 1:**
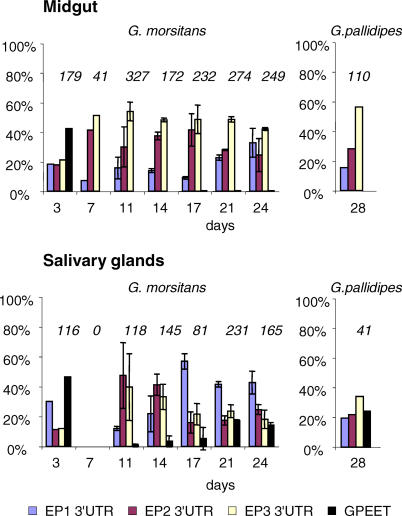
Distribution of Procyclin mRNAs Expressed by Tsetse-Derived Trypanosomes RNA was isolated from the midguts and salivary glands at various days post infection and subjected to RT-PCR using generic primers for all procyclins. The products were cloned and individual clones assigned by hybridisation with a panel of oligonucleotides (see Materials and Methods). The total number of procyclin clones analysed at each time point is indicated within the chart. The standard deviations for the data from days 11–24 derive from two independent tsetse infections.

At day 3, post-infection, the most abundant species of procyclin cDNA obtained from midgut trypanosomes was GPEET (42%). Based on their 3′ UTRs, each of the three EP isoforms accounted for 18–21% of the total. By day 7, however, no GPEET clones were obtained and EP3 and EP2 became the predominant isoforms (51% and 42%, respectively) followed by low levels of EP1 (7%). Very similar patterns of procyclin expression were found in midgut trypanosomes isolated up to 24 d post-infection. On the whole, this resembles the pattern of procyclin proteins present in these stages, with the exception that the EP2 isoform has never been detected in the fly. The stock AnTat 1.1 used in this study contains two hybrid genes [11]. Besides one normal copy each of EP1 and EP2, there are two genes in which the EP3 coding region is fused to the 3′ UTR of EP1 or EP2, respectively. The coding regions of EP1 and EP3 could not be distinguished by hybridisation because their open reading frames (ORFs) differ by only three nucleotides outside the repetitive region. In contrast, the coding regions of the clones that were positive for the EP2 3′ UTR could be assigned using specific oligonucleotides. We found that the EP2 and EP3 coding regions were equally represented (unpublished data) and that mRNAs encoding EP2 therefore constitute only ~15–20% of the total procyclin transcripts in midgut trypanosomes.

When salivary gland RNA was analysed, procyclin cDNAs could be amplified on day 3, but not on day 7. The PCR products obtained on day 3 were probably due to trypanosomes that entered the mouthparts during the initial infective blood meal and persisted there until the first meal of uninfected blood. Procyclin mRNAs were detected consistently in the salivary glands from day 11 onwards. This correlates with the first appearance of trypanosomes in this compartment [4]. Surprisingly, the relative amounts of the various PCR products changed during the course of infection. While the distribution on day 11 resembled that of established midgut forms, with 12% with an EP1 3′ UTR and only a single GPEET clone, over the following 2 wk there was an increase in both these forms, with the EP1 3′ UTR cDNAs finally accounting for 40% and GPEET for 16%. The differences in expression between midgut and salivary gland trypanosomes were reproducible in *G. morsitans morsitans* and were also apparent in another species of tsetse fly, *G. pallidipes* (Figure 1, right panels).

We next performed double immunofluoresence on trypanosomes from the salivary glands with antibodies directed against EP and GPEET. Midgut trypanosomes and cultured procyclic forms were used as controls. The majority of trypanosomes in the salivary glands did not react with either antibody, but we occasionally detected cells that were positive for EP and/or GPEET. EP-positive trypanosomes were detected slightly more frequently, but the fluorescence intensity was much weaker than that of midgut trypanosomes (unpublished data).

### Levels of Procyclin Transcripts in the Midgut and Salivary Glands

The RT-PCR experiments described above gave information on the distribution of the different procyclin transcripts in salivary gland trypanosomes, but not on their absolute amounts, and it could not be excluded that these were extremely low. We therefore used real time quantitative PCR to monitor the level of procyclin mRNAs in infected flies. Since variable amounts of tsetse RNA were present in the samples, procyclin transcripts were normalised against trypanosome β-tubulin. Values are expressed relative to a reference cDNA from cultured procyclic forms that was run in parallel with each set of samples, in which the procyclin/tubulin ratio was set at 1 (Figure 2). Throughout the course of infection, the cDNAs obtained from the midgut gave slightly higher levels of procyclin than the reference culture (1.6- to 3.9-fold). In agreement with previous estimates of procyclin transcripts in long slender bloodstream forms, we found that the level of procyclin mRNA was 22 times lower than in the reference culture and, on average, 53 times lower than in midgut forms. However, trypanosomes from the salivary glands contained substantially more procyclin mRNA than bloodstream forms. The samples taken from the salivary glands at days 11 and 14 contained slightly more procyclin mRNA than the reference culture (1.2–fold) and approximately half as much as midgut forms; as the infection progressed this declined to three times less than the reference culture and eight times less than midgut forms. This decrease may reflect the higher proportion of metacyclic forms, with correspondingly less procyclin mRNA, in mature infections.

**Figure 2:**
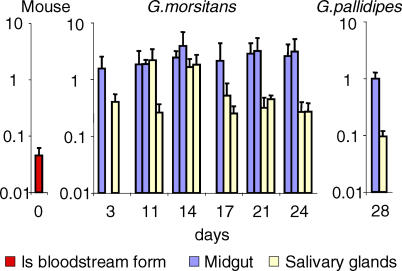
Relative Amount of Procyclin mRNA in Different Life Cycle Stages RTQ-PCR was performed on RNA samples from bloodstream forms and midgut and salivary gland trypanosomes at various days post infection. β-tubulin was used as an internal control. The ratio of procyclin/tubulin mRNAs in a reference culture of procyclic forms was set at 1. Standard deviations derive from two to five replicas. RNA derived from independent infections (days 11–24) was quantitated separately.

### Regulation of Expression by 3′ UTRs

If procyclin transcripts are present at relatively high levels, yet no proteins can be detected by immunofluorescence, it might be that post-translational modifications make them inaccessible to antibodies. It has been shown that a major surface glycoprotein of *T. congolense*, glutamic acid/alanine-rich protein (GARP), which is expressed by epimastigote forms [17] is decorated by oligosaccharides attached to threonine residues [18]. Should GPEET be similarly modified, it might not bind antibodies that are directed against a synthetic peptide [19] or phosphorylated GPEET [14]. To circumvent this problem we replaced one copy of GPEET with GPEEA, a mutant in which the threonine residues in the repeat have been substituted by alanines [20]. This mutation does not alter midgut infections [20]. Although procyclic cells expressed this mutant form of procyclin, we did not find more GPEEA-positive cells than GPEET-positive cells in the salivary glands (unpublished data).

An alternative explanation for the presence of transcripts, but lack of proteins, is that translation is regulated by the 3′ UTRs. To test this hypothesis we generated four mutants in which the GARP coding region replaced that of an endogenous procyclin. In this context, the mRNA is transcribed as part of a procyclin expression site and has the same UTRs as the corresponding procyclin. Flies were infected with individual mutants and GARP expression was monitored by immunofluorescence as described previously [3,15]. The majority of cells expressing the hybrid GARP/EP1 transcript, including post-mesocyclic and salivary gland forms, were positive by immunofluorescence (Figure 3). Apart from the earliest time point, cells expressing GARP/EP3 were positive throughout the midgut, as were 21% of salivary gland forms. In contrast, the midgut forms of GARP/EP2 mutant were negative for the first 7 d, and positive trypanosomes in the anterior midgut and salivary glands were detected in only two out of three flies. Trypanosomes expressing GARP/GPEET were positive early in infection and negative from day 7 onwards. However, 48% of the salivary gland trypanosomes were positive at day 28. Taken together, these results indicate that each 3′ UTR confers a unique expression profile. In all cases however, GARP can be translated in the salivary glands, indicating that the lack of the procyclin protein is not due to inhibitory sequences in the 3′ UTRs.

**Figure 3:**
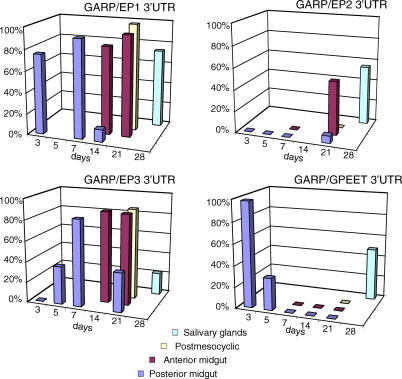
Effect of Procyclin 3′ UTRs on Expression of a Reporter Gene during Fly Transmission The coding region of GARP was fused to individual procyclin 3′ UTRs and stably integrated in place of the corresponding procyclin coding region. The percentage of trypanosomes expressing GARP in different compartments of the digestive tract and the salivary glands of the tsetse fly was determined by immunofluorescence analysis. For the sake of clarity the figures are set to the same scale. Blank spaces on the graphs indicate that no samples were taken from a particular tissue.

## Discussion

By analysing the spectrum of procyclin mRNAs expressed by *T. brucei* during the course of infection we have gained insights into the mechanisms regulating procyclin expression in the tsetse fly *G. morsitans*. At the beginning of an infection, GPEET mRNA is the predominant form in midgut trypanosomes, but is completely absent from late procyclic forms. In this respect, GPEET regulation operates the same way in the tsetse fly and in culture [3]. In late procyclic forms isolated from *G. morsitans*, approximately 85% of the mRNAs encode the glycosylated forms EP1 and EP3, and 15% encode EP2. When the distribution of proteins [12] and mRNAs from midgut trypanosomes are compared, the one disparity is that there is no EP2 protein, despite the relatively high level of its mRNA. Two lines of evidence indicate that the EP2 3′ UTR is responsible for inefficient translation in procyclic forms. When procyclic culture forms were transiently transfected with reporter constructs containing the chloramphenicol acetyl transferase (CAT) coding region, eight times less CAT activity was obtained when the construct contained the EP2 3′ UTR rather than the EP1 3′ UTR [11]. Furthermore, a stable transformant containing a GARP/EP2 hybrid gene showed no expression in the posterior midgut, although positive trypanosomes were detected in the anterior midgut and salivary glands.

As mentioned above, no procyclins could be identified in the salivary glands by MALDI-TOF MS. Nevertheless, procyclin transcripts were present at levels comparable to those in procyclic culture forms and cDNAs were readily amplified from the salivary glands. Apart from the samples taken at day 11, the time at which trypanosomes start to colonise the salivary glands, the distribution of procyclin mRNAs differed from that in the midgut, with increasing amounts of EP1 and GPEET mRNAs and decreasing amounts of EP2 and EP3 mRNAs as the infection aged. Since GPEET and EP1 are the first genes in the procyclin expression sites on chromosome VI and X, respectively, these differences may reflect an attenuation of transcription from the procyclin promoters in epimastigote or metacyclic forms, as is the case in bloodstream forms [21]. Alternatively, they may be due to changes in mRNA stability between midgut and salivary gland forms. The 3′ UTRs are known to have a major influence on the stability of procyclin transcripts [3,22–24] and it is worth noting that the 3′ UTRs of EP1 and GPEET are 86% identical.

Extremely few trypanosomes from the salivary glands or saliva reacted with antibodies against any form of procyclin. The most plausible explanations for these findings are that the mRNAs are not translated or that the proteins are rapidly degraded. Fixation with acetone exposes internal epitopes, so the failure to detect procyclins was not due to alterations in protein trafficking. It is also unlikely that the proteins were destroyed by proteases since the EP and GPEET repeats are extremely robust and resist digestion by tsetse midgut proteases [12,25] and the trypanosome's own surface protease(s) [26]. We could also exclude post-translational modifications such as O-linked carbohydrates for the lack of reactivity with anti-GPEET antibodies. While we cannot completely eliminate the possibility that the procyclin repeats are otherwise masked or modified by tsetse factors, shielding them from antibodies, it should not be forgotten that the tsetse also expresses a protein, tsetseEP, that contains an extended glutamic acid-proline repeat and that this protein can be detected in salivary gland lysates by the same monoclonal antibody that recognises EP repeats in procyclins [27].

In contrast to EP and GPEET proteins, GARP could be detected readily in salivary gland forms of transgenic trypanosomes, although the mRNA was transcribed from a procyclin promoter and the ORF was flanked by procyclin 5′ and 3′ UTRs. This clearly suggests that at this stage of trypanosome development in the tsetse fly, the procyclin coding regions are involved in regulating protein expression. We have previously encountered a similar phenomenon in bloodstream form trypanosomes that expressed high levels of EP1 mRNA (once negative elements were removed from the 3′ UTR) but no detectable protein [24] as long as they were maintained at 37 °C. It is worth noting, however, that this translational regulation appears to be lost in cold-shocked bloodstream forms [28].

In the light of these results, it will be necessary to revise the concept that all life cycle stages of *T. brucei* are covered either by VSG or procyclins as has long been supposed. The identification of alternative coats may prove challenging, however, as long as the only specific marker for the epimastigote form is the anteronuclear position of the kinetoplast and this form of the parasite can be obtained only from infected flies.

## Materials and Methods

### Trypanosome stocks.

The pleomorphic stock *Trypanosoma brucei brucei* AnTat1.1 [29,30] and mutants that derived from this strain were used in this study. For bloodstream forms, NMRI mice (Charles River Laboratories, France) were immunosuppressed by intraperitoneal (i.p.) injection of (200 mg kg^−1^ body weight) cyclophosphamide 6–12 h before i.p. injection of 10^6^ long slender bloodstream form trypanosomes. Short stumpy bloodstream forms were triggered to differentiate to procyclic culture forms in SM medium containing 20% foetal bovine serum [31] and cultivated at 27 °C. Alternatively, they were stimulated to differentiate by the addition of 6 mM *cis*-aconitate to the culture medium and a temperature shift to 27 °C [32]. Procyclic culture forms were maintained in SDM-79 [33] supplemented with 10% foetal bovine serum and 20 mM glycerol.

### Infection of tsetse flies with wild type AnTat 1.1

Pupae of *Glossina morsitans morsitans* and of *Glossina pallidipes pallidipes* were obtained from the International Atomic Energy Agency (Vienna, Austria) and the tsetse flies were maintained at the Swiss Tropical Institute, Basel, Switzerland as described elsewhere [19]. Short stumpy bloodstream forms (10^6^ ml^−1^) were fed to teneral flies with their first meal of horse blood; thereafter, infected flies were fed horse blood at 2–3 d intervals until dissection [3,19]. At days 21, 24, and 28 post infection, fly saliva was inspected microscopically for trypanosomes and the positive flies (three to seven per experiment) were used for RNA isolation. At earlier time points all flies (40–100 per RNA isolation) were used, without prior identification of flies with salivary gland infections.

### RNA isolation.

Long slender bloodstream forms were harvested from mouse blood at 3 d, and short stumpy forms at 5 d post infection. The blood was collected in 4 ml phosphate buffered saline (PBS: 137 mM NaCl, 2.7 mM KCl, 10 mM Na_2_HPO_4_, 1.76 mM KH_2_PO_4_, [pH 7.4]), prewarmed to 37 °C, containing 10 mM glucose and 100 U ml^−1^ heparin (Serva, Heidelberg, Germany). Trypanosomes were purified from the red cells by centrifugation (10 min, 1000 × g at room temperature) and isolation of the “buffy coat”. RNA from these purified bloodstream form trypanosomes was isolated using hot phenol [34]. RNA from procyclic culture forms, 5 d after differentiation, was isolated using an RNeasy Mini Kit (Qiagen, Hilden, Germany). RNA from infected tsetse tissue was isolated as follows: salivary glands, including the salivary ducts, were isolated by dissection and pooled in 1 ml of PSG (60 mM phosphate buffer, 40 mM NaCl, 1% glucose [pH 8]) with those of the other flies from the same experiment. The midguts from the same flies were isolated and pooled separately. The midgut tissue was disrupted mechanically and centrifuged for 5 s at 250 × g. The pellet was washed in 200 μl PSG and centrifuged again. Salivary glands and the supernatants of the midgut tissues containing the trypanosomes were centrifuged at 1,750 × g for 10 min. RNA was isolated from the pellets using a Strataprep Total RNA Microprep Kit (Stratagene, La Jolla, California, United States).

### cDNA preparation.

Total RNA (11 μl [≤1 μg]) was mixed with 10 pmol primer (GGCCACGCGTCGACTAGTAC(dT)_18_) and water to a total volume of 12 μl, incubated for 10 min at 70 °C and chilled on ice for 1 min. Then, 4 μl of first-strand buffer (Invitrogen, Carlsbad, California, United States), 2 μl of 0.1M DTT and 1 μl of dNTPs (10 mM each) were added and the reaction was incubated for 5 min at 42 °C. Superscript II H^−^ (Invitrogen, Carlsbad, California, United States) reverse transcriptase (1 μl [200U]) or 1 μl of water was added and incubated for 50 min at 42 °C followed by an incubation at 70 °C for 15 min.

### Identification of procyclin cDNAs.

Procyclin cDNAs were amplified from 2 μl of the cDNA reaction by PCR using *Taq* polymerase (Qiagen, Hilden, Germany) and a single primer pair corresponding to a conserved sequence in the 5′ UTR (CACAATGGCACCTCGTTCCCTTTATC) and the adaptor sequence added during reverse transcription (GGCCACGCGTCGACTAGTAC). The cycling conditions were as follows: 3 min, 94 °C; 30 × [1 min, 94 °C; 1 min, 65 °C; 1.25 min, 72 °C], 3 min, 72 °C. PCR products were cloned using a TOPO TA Cloning Kit (Invitrogen, Carlsbad, California, United States) and 100 μl of overnight cultures of individual clones were dot blotted onto nylon membranes (Roche, Rotkreuz, Switzerland). The bound DNA was denatured, neutralized and UV cross-linked according to standard procedures. The cloned procyclins were identified by sequential hybridization with oligonucleotides specific for the different EP 3′ UTRs and the GPEET ORF.

 EP1 3′ UTR: GGTCTCAGGCGATGGTTAATAAGGCATTAG

EP2 3′ UTR: CGTATATGCAAGTGTCCTGTCGCC 

EP3 3′ UTR: TTATGCGTGTCAGTGTCCTGTCACAAAGGA

GPEET ORF: AGGTTCAGTTTCCTCTGGTCCCGT 

Procyclins containing an EP2 3′ UTR were further analysed for the presence of an EP2 or EP3 ORF using the oligonucleotides EP2 ORF, GTTCAGGCTCAACTTCGTCCTG, and EP3 ORF, TCGCTGACCTTGGTTCCCTTCC. Oligonucleotides were end-labelled with ^32^P using polynucleotide kinase [35]. Hybridization was performed overnight with 0.2 nM labelled oligonucleotides at 37 °C in hybridization buffer (0.1% sodium pyrophosphate, 6 × SSC, 5 × Denhardts, 0.5% sodium dodecyl sulfate (SDS), 100 μg ml^−1^ salmon sperm DNA) followed by two 30-min washes in 1 × SSC, 0.05% SDS at 50 °C.

### Real time quantitative PCR.

Real time quantitative PCR (RTQ-PCR) was performed on 0.12 μl of the cDNA reactions in 30 μl of Platinum Quantitative PCR SuperMix-UDG (Invitrogen, Carlsbad, California, United States) with the primers (800 nM) and TaqMan probes (100 nM) described below. Procyclin cDNA (all isoforms) was measured with the oligonucleotides CTGTACTATATTGACTTCAATTACACCAAAAAG, AGGTTCGCGCTGAACAGAAG and FAM-TTCACAATGGCACCTCGTTCCCTTTATC-TAMRA. *T. brucei* β-tubulin cDNA was measured with the oligonucleotides ACTGGGCAAAGGGCCACTAC, CTCCTTGCAGCACACATCGA, and FAM-CGGAGGGTGCGGAACTGATCGAC-TAMRA. The average amplification efficiencies were 1.85 for the procyclin RTQ-PCR and 1.9 for the β-tubulin RTQ-PCR. The reactions were carried out with the Gene Amp 5700 Sequence Detection System from Perkin Elmer Biosystems (Boston, Massachusetts, United States) and analysed with its software version 1.3 using the standard protocol. Each run was calibrated with a standard cDNA from procyclic culture forms. The amount of procyclin or tubulin cDNA in a sample could be calculated from the relative standard curve as an n-fold difference from the standard cDNA. The results of the procyclin RTQ-PCR were then normalized to the internal control, the tubulin RTQ-PCR. Experiments were repeated three to six times to calculate the standard deviations.

### Constructs and transgenic trypanosomes.

GARP/EP1, GARP/EP2, GARP/EP3 and GARP/GPEET were designed to replace the coding region of the relevant procyclin gene precisely with that of the GARP gene, upstream of the given 3′ UTRs [23]. The constructs were named according to the procyclin 3′ UTR flanking the GARP gene. Each plasmid also contains an antibiotic-resistance gene for selection of stable transformants. These constructs are derivatives of CAT/EP1, CAT/EP2, CAT/EP3 and CAT/GPEET, respectively [11], in which the CAT gene was released by cleavage with HindIII and BamHI and replaced by the GARP coding region [23]. For stable transformation, GARP/EP1 and GARP/EP2 were linearized with KpnI and NotI and GARP/EP3 and GARP/GPEET were linearized with SalI and XbaI. GARP/EP1, GARP/EP3 and GARP/GPEET were used for stable transformation of procyclic forms of AnTat 1.1. This was performed as described for stock 427 [3], except that transfected cells were resuspended in SDM-79 supplemented with 30% conditioned medium and 5 × 10^5^ untransformed cells per ml medium. GARP/EP2 was used for stable transformation of bloodstream forms of AnTat 1.1 as described [36]. Fly transmission of transgenic procyclic forms to produce bloodstream forms was performed essentially according to Jefferies et al. [37].

### Analysis of GARP expression during trypanosome development in the tsetse fly.

Teneral *G. morsitans morsitans* males, 8–32 h after emergence, were given a first, infective bloodmeal from immunosuppressed NMRI mice that showed a parasite load of more than 5 × 10^8^ ml^−1^ trypanosomes of one of the GARP-tagged clones. Infected flies were further maintained on uninfected rabbits until 48 h before dissection.

Flies were dissected at different times after the infective blood meal: days 3, 5, 7, 14, 21, and 28. Depending on the progression of infection in the tsetse, trypanosome populations were collected from different sites in the fly: (i) procyclic trypanosomes from the posterior midgut, (ii) mesocyclic trypanosomes from the anterior midgut near the proventriculus (part of the midgut between the mycetome and the proventriculus), (iii) post-mesocyclic trypanosomes from the proboscis, and (iv) trypanosomes from the salivary glands.

For each sample point, trypanosomes from three positive flies were individually collected on a glass slide in a drop of PBS. These slides were air-dried and trypanosomes were fixed with acetone for 10 min at −20 °C. They were stored dry over silica gel at −20°C until analysis by immunofluorescence.

To detect GARP expression, immunofluorescence was performed with a rabbit polyclonal antiserum (diluted 1:500) raised against a GARP-glutathione S-transferase fusion protein [3]. EP procyclin expression was detected with the monoclonal antibody (mAb) TRBP1/247, diluted 1:400 [38], and GPEET with a rabbit polyclonal antiserum (K1; [19]) or mAb 5H3 [14], both diluted 1:400. The mAbs were kindly provided by Terry W. Pearson (University of Victoria).

## Abbreviations

CAT: chloramphenicol acetyl transferase
GARP: glutamic acid/alanine-rich protein
mAb: monoclonal antibody
MALDI-TOF: matrix-assisted laser desorption time-of-flight
ORF: open reading frame
RTQ-PCR: real time quantitative PCR
UTR: untranslated region
VSG: variant surface glycoprotein
